# Going Beyond Conventional Mammographic Density to Discover Novel Mammogram-Based Predictors of Breast Cancer Risk

**DOI:** 10.3390/jcm9030627

**Published:** 2020-02-26

**Authors:** John L Hopper, Tuong L Nguyen, Daniel F Schmidt, Enes Makalic, Yun-Mi Song, Joohon Sung, Gillian S Dite, James G Dowty, Shuai Li

**Affiliations:** Centre for Epidemiology & Biostatistics/Melbourne School of Population & Global Health, Faculty of Medicine, Dentistry & Health Sciences, The University of Melbourne, Victoria 3010, Australia; nguk@unimelb.edu.au (T.L.N.); daniel.schmidt@monash.edu (D.F.S.); emakalic@unimelb.edu.au (E.M.); yunmisong@skku.edu (Y.-M.S.); jsung@snu.ac.kr (J.S.); g.dite@unimelb.edu.au (G.S.D.); jdowty@unimelb.edu.au (J.G.D.); shuai.li@unimelb.edu.au (S.L.)

**Keywords:** breast cancer, cumulus, cirrocumulus, cirrus, mammogram-based risk, mammographic density, OPERA

## Abstract

This commentary is about predicting a woman’s breast cancer risk from her mammogram, building on the work of Wolfe, Boyd and Yaffe on mammographic density. We summarise our efforts at finding new mammogram-based risk predictors, and how they combine with the conventional mammographic density, in predicting risk for interval cancers and screen-detected breast cancers across different ages at diagnosis and for both Caucasian and Asian women. Using the OPERA (odds ratio per adjusted standard deviation) concept, in which the risk gradient is measured on an appropriate scale that takes into account other factors adjusted for by design or analysis, we show that our new mammogram-based measures are the strongest of all currently known breast cancer risk factors in terms of risk discrimination on a population-basis. We summarise our findings graphically using a path diagram in which conventional mammographic density predicts interval cancer due to its role in masking, while the new mammogram-based risk measures could have a causal effect on both interval and screen-detected breast cancer. We discuss attempts by others to pursue this line of investigation, the measurement challenge that allows different measures to be compared in an open and transparent manner on the same datasets, as well as the biological and public health consequences.

## 1. Introduction

This Special Issue calls for manuscripts that will help advance our understanding of mammographic density to improve the detection and prevention of breast cancer. But the issue is much larger than mammographic density, at least as it has been defined conventionally as the ‘white or bright areas on a mammogram’. The last few decades had seen many efforts to measure this historical concept, including automated measures now that digital mammography is widespread, building on the seminal work of Boyd and Yaffe who created a semi-automated method [1]. But those efforts had missed a fundamental point. The aim shouldn’t be to better measure mammographic density *per se*, but to better predict risk.

This commentary is about finding ways to predict a woman’s breast cancer risk from her mammogram, building on the work of Wolfe, Boyd and Yaffe on what has become known as mammographic density. We summarise our efforts at finding new mammogram-based risk predictors, and how they combine with the conventional measure of mammographic density in predicting breast cancer risk for interval cancers and screen-detected cancers, as well as across different ages at diagnosis and for both Caucasian and Asian women. We also discuss more recent attempts by others to pursue this line of investigation, the Measurement Challenge that allows different measures to be compared in an open and transparent manner on the same datasets, as well as the biological and public health consequences.

Before we do so, we discuss the pivotal concept of OPERA (odds ratio per adjusted standard deviation) which should not be relegated to an Appendix or Supplementary Material. It is fundamental to understanding, and putting into proper perspective, the relative strengths of risk factors with one another, and with other risk factors. It is particularly relevant to mammographic density due to its negative confounding with the major breast cancer risk factor of age and, for some, another breast cancer risk factor in body mass index. The reader might want to first skip this section and go straight to the section on Wolfe and beyond, but it is essential to understanding the area. 

## 2. OPERA: A Measure of the Ability of a Risk Factor to Identify Future Cases on a Population Basis

Consider the issue of how to rank risk factors in terms of their ability to predict disease risk on a population-basis; namely the extent to which they differentiate cases from controls. For simplicity, we consider risk factors measured on a continuous scale, though this is not an essential assumption. The key issue is to recognise that disease risk almost invariably depends on age, and often sex (dramatically so for breast cancer), as well as many other factors. Some of these factors are measured and adjusted for in analysis while others can be controlled for, at least to some extent, by design. Consequently, studies of breast cancer and mammography measures typically consider women only, and use cases and controls who are similar, or even matched for, age. Some family-based designs control for familial factors—genetic and environmental. The result is an estimate of relative, not absolute, risk—the latter can be determined by appropriate use of population age-specific incidences. 

The point that is often not appreciated is that the resulting risk estimate applies to a change in the risk factor of concern *while holding all other factors constant*. Therefore, the risk scale is not that of the unadjusted risk factor. Expressing the risk gradient in terms of units of the *unadjusted* risk factor is inappropriate, yet standard practice throughout epidemiology despite it giving misleading results.

For mammogram-based risk measures in particular, these considerations are particularly important. Conventional mammographic density declines with age in the decades around the menopausal years. Percent mammographic density has a mean of around 25–35% before age 40 years, then decreases over the next two decades to plateau at about 15–25% in later life for Caucasian women [2]. A similar pattern with age has been observed for Asian women [3]. After adjusting for age and body mass index, percent density has a standard deviation of ~10% and is highly, though not completely, stable (correlation between measures 10 years apart ~ 0.9) [2].

The commonly used concept of percent mammographic density (absolute density divided by the total breast size) is also moderately, and negatively, correlated with body mass index. But breast cancer risk increases substantially with age, and post-menopause, with body mass index. Intriguingly, body mass index has a negative (i.e., protective) association with breast cancer risk prior to menopause [4]. Consequently, almost all studies of mammogram-based risk measures use case-control samples of similar ages, and adjust for age, body mass index, and often other measured risk factors though the latter don’t explain much variance [5]. The fact that the adjustment has been made in the analysis is typically denoted in the footnotes to Tables of results but rarely commented on in the text.

Major problems arise when the estimated risk gradient is presented in terms of the so-called ‘change in risk per standard deviation’ specifically as the increment in risk for a one-unit change of the standard deviation of the unadjusted, raw measures [6]. These ‘per standard deviation’ risks are not meaningful (they do not represent what has been estimated) and should not be used to compare risk factors across measures.

The considerations above when applied to mammogram-based risk factors were the driving force in us developing the concept of OPERA (odds ratio per adjusted standard deviation) [7]. The word “adjusted” refers to the use of the standard deviation of the risk factor (for the population about which inference is being made) after it has been *adjusted* for other factors being taken into consideration by the design *and* analysis. 

OPERA is the correct way to interpret risk estimates in terms of risk discrimination on a population basis because it recognises that a risk factor is in effect the risk measure adjusted for covariates, not the raw measure itself. In addition, if it is assumed that the risk measure is approximately normally distributed (typically researchers have transformed mammographic density measures to achieve this) and that risk increases multiplicatively across the risk spectrum (which empirically appears to be a reasonable assumption), then the log(OPERA) is approximately equal to the difference in means (in terms of standard deviation for the population) between cases and controls, and so is easily interpreted. 

The measure log(OPERA), therefore, is an appropriate scale on which to discuss and compare risk discrimination. There is a mathematical relationship between log(OPERA) and the area under the receiver operating characteristic curve (AUC) which is approximately linear at least for AUC between 0.5 to 0.7 (OPERA between 1 and 2). Under these assumptions, the inter-quartile risk ratio (IQRR) is approximately OPERA^2.5^ (see Supplementary Material [8]). 

When OPERA is used, it emerges that conventional mammographic density is one of the stronger risk factors for female breast cancer. The OPERA is typically around 1.4, substantially more than for the classic epidemiological risk factors, as discussed previously [7]. The risk discrimination from conventional mammographic density compares favourably with that achieved by the latest polygenic risk scores for which the OPERA is about 1.6 [9]. Our new mammogram-based risk measures have OPERAs of 1.6 or higher [8,10,11,12,13,14] and we will now discuss how this came about.

## 3. Wolfe and Beyond 

In the 1970s, John Wolfe demonstrated that certain aspects of a mammogram were predicting, for women deemed at a mammographic screen to not have a breast cancer, which of them were more likely to be diagnosed with breast cancer at subsequent screens [15]. An advocate of early mammographic screening, Wolfe developed a classification scheme that initially apparently failed to gain adoption by radiologists due to lack of replication in measurement and inconsistent results [16], though the latter might have been a consequence of the former. It was recognised, however, that the dense regions of mammograms were implicated in a major weakness of mammographic screening-the failure to detect cancers hidden, or ‘masked’, by the white or bright regions that are mammographically ‘dense’.

The seminal work of Boyd and Yaffe, who created a semi-automated way to measure mammographic density called CUMULUS [17] and applied it to prospective studies of large screening populations, consistently found evidence that percent mammographic density—adjusted by design and/or analysis for age and body mass index—was predicting both interval and screen-detected cancers in the long-term [18]. Use of the OPERA concept confirmed that this risk gradient was stronger for interval cancer [19], consistent with a role of masking. For example, Krishnan and colleagues [20] found that for percent density adjusted for age and body mass index, the OPERA was 2.05 (95% confidence interval (CI) 1.65 to 2.56) for interval cancer compared with 1.37 (95% CI 1.21 to 1.56) for screen-detected cancer (*p* for difference = 0.002). Moreover, percent mammographic density was the best predictor of whether a woman was to develop interval versus screen-detected cancer, with an OPERA of 1.51 (95% CI 1.23 to 1.86; *p* = 0.00005).

Therefore, percent mammographic density was clearly predicting something specific to interval cancers, presumably masking, and was also associated with intrinsic breast cancer risk, but to a far lesser extent. While its association with interval cancer was by its nature likely to be causal, in the sense that changing percent density could lead to a reduction in risk, it was still unclear if the conventional measure of density, or even density per se, is causal for breast cancer. It is possible that the conventional concept of density as a risk factors is correlated with other mammogram-or lifestyle-related causal risk factors. To examine these hypotheses, we took two approaches to discovering new mammogram-based measures of risk, and importantly, considered their risk predictions concurrently with those arising from conventional mammographic density when fitted together.

## 4. Redefining Mammographic Density in Terms of Brightness: *Cumulus, Altocumulus* and *Cirrocumulus*

Initially, mammographic density was defined as the light or bright area and calculated using the CUMULUS software [17]. We call the resultant measure *Cumulus* in honour of the method and its creators, and because it aligns with the meteorological use of this word to describe clouds often described as puffy, cotton-like or fluffy and based on the Latin word “cumulo”, meaning heap or pile. 

We challenged this convention by defining mammographic density at, in effect, two substantially higher pixel-brightness thresholds as the brighter (*Altocumulus*) and brightest (*Cirrocumulus*) regions. We used the CUMULUS software but chose the bright regions within the white areas to define *Altocumulus*, and the brighter regions among the bright areas to define *Cirrocumulus*. These words also align with their meteorological use by matching pixel intensity to height (the Latin word “alus” means high). Altocumulus are middle altitude clouds typically higher than cumulus clouds and have elements that are larger than those of cirrocumulus clouds which typically appear at an even higher altitude—the Latin word “cirro” means curl and is used to describe a high cloud.

Operators of CUMULUS have traditionally been trained to be highly repeatable in their measures of *Cumulus*, both with themselves and with other operators. We similarly trained operators to ensure measures of *Altocumulus* and *Cirrocumulus* were highly repeatable.

We found from multiple studies of Australian and Korean women [10,11,12,13,14] that the new measures of mammographic density, *Altocumulus* and *Cirrocumulus*, did better than *Cumulus* at predicting risk. One notable exception is risk of interval breast cancer [14]. 

Table 1 and Table 2 show that the risk gradients for *Altocumulus* and/or *Cirrocumulus* were comparable to those for the genetic risk scores developed from genome-wide association studies (OPERA = 1.6; [9]) and often better than for *Cumulus* (OPERA = 1.4). The risk estimates for *Altocumulus* were often little different to those for *Cumulus*, but the risk estimates for *Cirrocumulus* in particular were in general stronger, especially for Korean women (who are diagnosed on average a decade or so earlier than Australian women) [10] and for Australian women diagnosed at a younger age [11]. These findings applied to film and well as digital mammograms, and for the latter, appeared to depend on the manufacturer [12].

## 5. Revisiting Texture using Agnostic Machine Learning: *Cirrus*

Wolfe originally described ‘parenchymal patterns’ using verbal descriptions and subjective measures of textural features [15]. We instead used machine learning to try to find agnostically if there are textural patterns (defined mathematically) that predict breast cancer risk [8]. We studied film mammograms from two samples of Australian women and from one sample of American-Hawaiian women living in Hawaii. We measured 20 textural features and found that the major principal component was based on 11 of these features. Risk measures created from these 11 features and learnt on one sample did well in predicting breast cancer in another sample (OPERAs ~ 1.7), even when learnt on the small sample of Japanese women and applied to Australian women. 

We call the risk measure based on fitting these 11 features to the combined sample *Cirrus*. These features were highly correlated with one another, and the best predicting individual feature was ‘homogeneity’, a measure of the degree of “scatteredness” of the texture within an image; images with large areas of similar intensity pixels have a higher degree of homogeneity than those composed of a large number of small dissimilar regions.

## 6. What Happens When the New Measures are Fitted Together with the Conventional Measure?

We now consider what happens when the new measures are fitted with and without taking into account the conventional measure, and *vice versa*. This allows inference to be made about the extent to which associations are due to confounding with one another from examining what happens to the estimates.

### 6.1. Cirrocumulus and Altocumulus

Except for interval cancer, when the new measures *Cirrocumulus* and *Altocumulus* were fitted with the conventional measure *Cumulus*, the *Cumulus* risk estimate attenuated towards the null and was often no longer statistically significant. In contrast, the new measures generally remained significant. This happened consistently, despite these measures all being correlated with one another (e.g., ~0.8 for *Cumulus* and *Altocumulus*; ~0.6 for *Cumulus* and *Cirrocumulus*). These general findings have been replicated [21]. 

Table 1 shows an example in which the different density measures (in absolute terms) were considered together as predictors of, on average, younger age at diagnosis breast cancer for women with a family history of breast cancer [11]. All three measures gave strong and somewhat similar risk predictions, with OPERAs of about 1.6–1.7, when considered alone (univariable analyses). When fitted together, the *Altocumulus* and *Cirrocumulus* measures both attenuated to be about 1.4 but remained highly significant. (The average of these two measures had an OPERA of about 1.8). The *Cumulus* measure, however, went to the null and was no longer significant. This happened for each combination that included *Cumulus*. The dense area measures gave a better fit than the percent mammographic density areas for which a similar pattern of results were observed (the univariable association for *Cumulus* as a percentage had a smaller OPERA of 1.52 (95% CI 1.34–1.73)).

Table 2. shows another example, this time for screen-detected breast cancer [13]. All three measures predicted risk on their own, with the risk gradient for *Cirrocumulus* being 24% greater on the log(OPERA) scale. When *Cirrocumulus* was fitted, the *Cumulus* measure went to the null and was no longer significant whether or not *Altocumulus* was also fitted.

Table 3 shows an example for interval breast cancer [13]. This time the percent mammographic density measures gave better fits than did the dense area measures. All three measures predicted risk on their own, with the risk gradient for *Cumulus* being the greatest. Contrary to the previous examples, it was the *Cumulus* measure that remained significant in the multivariable analyses whereas both *Altocumulus* and *Cirrocumulus* were no longer significant once *Cumulus* was also fitted.

### 6.2. Cirrus

For *Cirrus*, Table 4 shows what happened when *Cirrus* was fitted together with absolute and percentage *Cumulus* measures [8]. The *Cirrus* risk estimate remained high and attenuated by only 16% on the log(OPERA) scale, while the *Cumulus* risk estimate attenuated by more than 50%. Most of the data used these analyses came from a prospective cohort study that involved about 70% screen-detected and 30% interval cancers. Note that the approach used to create *Cirrus* only used relative brightness between pixels, not the absolute brightness of pixels as does the CUMULUS-based measures above.

## 7. Some Other New Mammogram-Based Measures of Breast Cancer Risk

Other researchers have also been trying to find aspects within a mammogram, other than mammographic density, that contain information about risk of breast cancer. But rarely have these researchers considered their new mammogram-based measure together with the conventional measure of mammographic density as we have done. 

One recent example is by Dembrower and colleagues [22] who developed a new risk measure using deep learning, which they call DL. Their DL measure was “moderately” correlated with conventional mammographic density measures (Spearman correlations between 0.25 and 0.42). Using a prospective cohort design, they combined interval and screen-detected cancers as outcome (278 cases and 2005 controls not matched for age, average 3.6 years follow-up, mean age at baseline mammogram 55 years) and calculated the OR per unadjusted standard deviation; see Table 5. They used LIBRA automated software [23] to calculate conventional dense area and percentage density and adjusted the mammogram-based measures for age (but not for body mass index, which might explain the poor predictive performance of their percent mammographic density measure, as is predicted by the earlier discussion about the OPERA concept). 

Table 5 shows that the risk gradient for the DL measure was unchanged by adjusting for the conventional mammographic density measures. The risk gradient for the absolute dense area measure halved after adjusting for the DL measure. Wanders and colleagues also considered together a conventional mammographic density measure and a texture method assessment method they had developed [24], but they adjusted the texture measure for mammographic density (but not vice versa). The risk gradient for the texture measure attenuated by about 15–35%, and remained highly significant. Unfortunately, they did not report what happened to the mammographic density risk after adjustment for texture.

## 8. Interpretation

**Figure 1:**
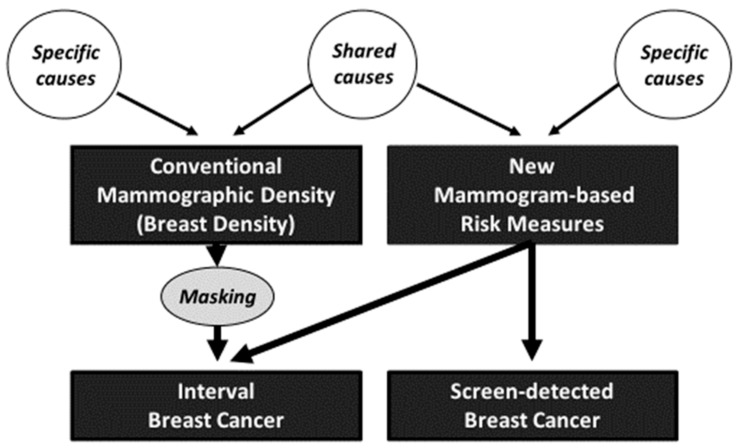
Path diagram indicating causal pathways implicated in conventional and new mammogram-based predictors of interval and screen-detected breast cancers.

Figure 1 shows an interpretation, in terms of a path diagram, of the general findings above. Unmeasured causes are shown as circles and the arrows represent causes in a given direction. The conventional and new measures are moderately correlated, so there must be causal factors specific to both measures (or even a causal relationship between the conventional and new measures, though we haven’t shown that on the figure). 

Because there is no convincing evidence that conventional mammographic density predicts screen-detected breast cancer once the other mammogram-based risk factors are taken into account, it appears to be a predictor of interval cancers due only (or mostly) to its role in masking. The new mammogram-based risk measures appear to be predicting interval cancer through their association with conventional mammographic density, particularly the other mammographic density measures defined by higher pixel brightness thresholds which attenuate once the conventional measure is fitted (see Table 3). But the new measures could also be having a direct effect given that their risk association is still evident even after adjustment for conventional mammographic density (see Table 1 and Table 2 and Table 4 and Table 5). The data are consistent with the new mammogram-based risk measures (rather than conventional mammographic density) having a direct causal effect on both interval and screen-detected breast cancer, and this hypothesis now needs to be addressed. 

## 9. Importance of Mammogram-Based Risk Factors as Breast Cancer Risk Predictors

**Figure 2:**
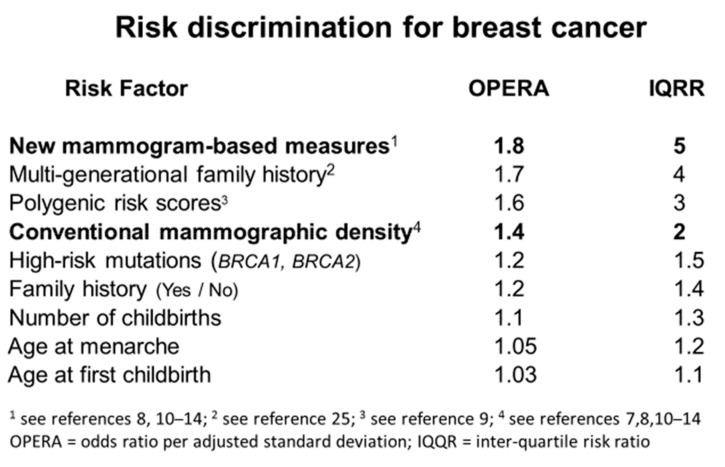
Risk discrimination for breast cancer based on the odds ratio per adjusted standard deviation (OPERA) (see [7]) and equivalent inter-quartile risk ratio (IQRR).

Following the concept of OPERA, Figure 2 shows that the new mammogram-based measures are the strongest of the currently known risk factors in terms of risk discrimination on a population-basis. This Figure is an update of Table 1 in the original paper on OPERA [7]. The next best risk factor is derived from multi-generational family history using the tools BOADICEA and BRCAPRO; the OPERA in Figure 2 pertains to risk of breast cancer diagnosed before age 50 years for women under the age of 50 years [25]. Given familial risks are highly dependent on age at diagnosis, and age at risk, this is likely to be greater than it would be for breast cancer diagnosed at any age. The OPERA for polygenic risk scores is based on the latest publication from the Breast Cancer Association Consortium [9]. 

Conventional mammographic density, adjusted for age and body mass index, is at most weakly associated with the other known lifestyle-based breast cancer risk factors [5], as mentioned in Section 1. Familial and genetic factors are perhaps the strongest; about 10–15% of the increased risk of breast cancer associated with having at least one affected first-degree relative family history is explained by the fact that mammographic density adjusted for age and body mass index has a correlation of about 0.3 in first-degree relatives and 0.6 in monozygotic twin pairs [5,26,27] Indeed, a similar proportion of the known genetic variants associated with breast cancer risk are also implicated in explaining the variance of mammographic density for age and body mass index [28,29]. The relationships between the new mammogram-based risk factors and known breast cancer risk factors have not yet been published.

## 10. Discussion

Wolfe was right, and wrong. There are aspects of a mammographic image that predict risk of breast cancer—but it isn’t all about parenchymal patterns, breast density, or the percentage of the breast covered by white areas. Wolfe’s initial strong predictions were, on face value correct, but they were mostly due to the strong role of conventional mammographic density in predicting masking, not so much as risk per se. 

Risk of interval cancers is an important issue, but it is not all due to density and masking. We have found that it can be greatly improved by treating density as a continuum (not as a binary construct as in current clinical practice) and by including other risk factors such as family history, and a new measure based on breast tissue ageing [14]. It will likely improve with the addition of polygenic risk scores and the other mammogram-based risk factors related to risk *per se*.

Our work has successfully identified new insights into breast cancer risk, but we would not have been able to do this without the enormous contributions of others. In particular, Professor Norman Boyd kept alive the ideas of Wolfe by developing the technology and resources, and inspiring the workforce, to pursue this line of investigation. The CUMULUS software has been an incisive tool for understanding breast cancer risk. We trained observers, not necessarily radiologists or physicians, to use CUMULUS in novel ways and achieved high repeatability and better prediction of breast cancer than the latest polygenic risk scores found from a massive expenditure on genetics applied to very large-scale epidemiological studies. These new mammogram-based risk factors are also much better than the other known epidemiological risk factors (see Figure 2) and complement pedigree-based risk models like BOADICEA and BRCAPRO [25].

What differentiates our work over the last few years has been its focus on the aspects of a mammographic image that predict risk, rather than on mammographic density alone. We have not been alone in this pursuit, at least not recently, and now that digital mammography is widespread, the prospect of finding even stronger risk predictors using agnostic computer-learning approaches is a reality. 

For example, Perutz and colleagues created OpenBreast [30], by applying logistic regression analyses to multiple features as we did in creating *Cirrus*, this time using digital mammograms but with a much smaller dataset. They recently reported that OpenBreast had an OPERA of 2.5, equivalent to an extraordinarily high IQRR of almost 10 but with a wide 95% CI (given the small dataset) for which the lower bound was 4 [30]. From the Supplementary Material [30], the most prominent feature contributing to Open Breast was ‘maximum gray-level value’, which could be picking up the aspects captured by *Cirrocumulus* (the amount of brightest areas). The next most prominent feature was ‘high gray-level run’ followed by ‘run-percentage’ and then both ‘short-run emphasis’ and ‘long-run emphasis’, separated by ‘gray-level range’. Therefore, OpenBreast could be utilising aspects of ‘homogeneity’ and the major principal component on which *Cirrus* is based, combined with measures of mean and range of brightness implicated in *Cirrocumulus*.

It might be that, by applying machine learning to digital mammograms, essentially the same risk-associated aspects of a mammogram are being discovered, but with greater power and more precision. A similar approach to identify texture-based risk predictors agnostically also performed well with an IQQR of about 6, with a lower bound of the 95% CI < 4 (again reflecting the small data set) [31]. 

Nevertheless, these two recent studies, combined with our discoveries using mostly film mammograms, are very encouraging for finding very strong, and easily automated, breast cancer risk prediction from applying machine leaning to digital mammograms. Our studies were detecting signals despite considerable noise from digitising film mammograms across different machines. Despite this, we appear to have been detecting true signals. Discovery of more and better signals might be much easier using digital mammography.

Another attempt to achieve this aim using ‘deep learning’ applied to a large data set [32] failed to use cases and controls of the same age, so they in effect learnt how to measure age from a mammogram, derived inflated estimates of risk discrimination based on naïve use of AUC statistics, and might not have discovered anything new. We know age predicts breast cancer risk, and is easily measured, so we want to know what it is about a mammogram that predicts risk for women of the same age. Reuse of that large data set to apply learning and replication using case-control sets matched for age could be worthwhile, especially if it was made openly available.

To help achieve the aim of determining the optimal measures of the different aspect of breast cancer risk from different mammographic images, we and others have created the Measurement Challenge [33]. This is an open international resource which offers sets of anonymised full-field digital mammogram images for analysis so that unbiased and transparent comparisons can be made between measures.

These new discoveries have important implications for understanding the causes of breast cancer. We have applied a new method for making inference about causation by examining familial confounding, called ICE FALCON [34], to data on twin pairs. We found no evidence that conventional DNA methylation in blood is having a causal effect on conventional mammographic density (*Cumulus*), or vice versa [35]. We will apply this approach to study our new mammogram-based risk factors. 

That conventional mammographic density might not necessarily be having a direct causal effect on breast cancer risk has serious implications for biological research. At this stage it is very difficult to identify which components of breast tissue are implicated in mammogram-based risk, but this will be an area of likely productive aetiological research once the new generation of risk factors emerging from agnostic approaches to digital mammograms are established and automated.

There are also major public health implications, especially given the magnitude of breast cancer over-diagnosis associated with mammographic screening [36]. It is interesting to reflect on Wolfe being an advocate of early mammographic screening. These new findings play to that scenario by making it possible to clarify with considerable precision, and at a young adult age, a woman’s risk of breast cancer [37]. 

If screening was to be tailored to a woman’s risk using new mammogram-based measures, family history and polygenic risk scores, large proportions of the population would be classified as well below ‘average’ risk while small proportions (but substantial numbers given the number of women screened) of women with risks as high as *BRCA1* and *BRCA2* mutation could be readily identified [37]. New automated risk predictors based on mammograms, improved genetic risk prediction and proper collection and utilisation of family history data could greatly change the landscape of breast cancer risk prediction without needing to collect data from women about lifestyle and other factors with small impact on risk discrimination, making implementation far more practical and realistic.

These advances in breast cancer risk prediction based on aspects of mammograms could also be used to help alleviate racial and ethnic disparities. For example, in the U.S., black women are more likely to be diagnosed with more aggressive interval breast cancer. Asian women also have on average higher percent mammographic density and are at increased risk of this type of breast cancer. Better risk prediction for interval breast cancer using multiple risk factors as in [14] is, therefore, a priority. To be effective, however, it will need to be automated, include new measures such as *Cirrus*, and be validated for use in racially and ethnically diverse populations. 

In summary, our research has directly challenged the historical paradigm of mammographic density research that there is one concept of mammographic density, defined as the light of bright regions on a mammogram, and that this measure is predictive of both masking of existing tumours and risk. We have drastically changed the conventional view by finding evidence that the conventional density is implicated in breast cancer risk—specifically interval cancers—by masking, not directly with risk per se. Other aspects of a mammogram, based on brightness and texture, appear to be more directly implicated in risk, and this applies to Caucasian as well as Asian women. The prospect of automated mammogram-based risk measures, combined with other major risk factors, could change population screening to be based more on relative risk than just age, make screening more effective, reduce its negative aspects, and hopefully save more lives.

## Figures and Tables

**Table 1:** Odds ratio per unadjusted standard deviation (OPERA^1^) for dense area mammographic density measures in predicting younger age at diagnosis breast cancer for women with a family history of breast cancer (Nguyen et al. (2017)) [11].

	Univariable	Multivariable	
*Cirrocumulus*	1.73 (1.52–1.97)	1.41 (1.21–1.65)	1.44 (1.20–1.72)
*Altocumulus*	1.74 (1.53–1.99)	1.43 (1.22–1.68)	1.48 (1.21–1.81)
*Cumulus*	1.62 (1.42–1.83)	–	0.95 (0.76–1.18)

^1^ standard deviation is calculated for each measure adjusted for age and body mass index using the controls sample and, for the multivariable analysis, in effect adjusted for the other measure.

**Table 2:** Odds ratio per unadjusted standard deviation (OPERA^1^) for dense area mammographic density measures in predicting screen-detected breast cancer (Nguyen et al. (2018)) [13].

	Univariable	Multivariable	
*Cirrocumulus*	1.32 (1.18–1.48)	1.19 (1.06–1.34)	1.19 (1.04–1.36)
*Altocumulus*	1.25 (1.12–1.40)	–	0.99 (0.85–1.14)
*Cumulus*	1.25 (1.12–1.40)	0.98 (0.87–1.10)	0.99 (0.85–1.15)

^1^ standard deviation is calculated for each measure adjusted for age and body mass index using the controls sample and, for the multivariable analysis, in effect adjusted for the other measure.

**Table 3:** Odds ratio per unadjusted standard deviation (OPERA^1^) for percent mammographic density measures in predicting interval breast cancer (Nguyen et al. (2018)) [13].

	Univariable	Multivariable	
*Cirrocumulus*	2.06 (1.67–2.54)	–	1.10 (0.90–1.36)
*Altocumulus*	1.92 (1.55–2.36)	0.88 (0.72–1.07)	-
*Cumulus*	2.33 (1.85–2.92)	1.67 (1.36–2.07)	1.48 (1.19–1.95)

^1^ standard deviation is calculated for each measure adjusted for age and body mass index using the controls sample and, for the multivariable analysis, in effect adjusted for the other measure.

**Table 4:** Odds ratio per unadjusted standard deviation (OPERA^1^) from Schmidt et al. combined data set combined data set [8].

	Univariable	Multivariable	
*Cirrus*	1.90 (1.73–2.09)	1.76 (1.59–1.95)	1.74 (1.57–1.93)
*Cumulus* (absolute)	1.34 (1.25–1.43)	1.16 (1.08–1.24)	–
*Cumulus* (percent)	1.38 (1.29–1.48)	–	1.16 (1.07–1.25)

^1^ standard deviation is calculated for each measure adjusted for age and body mass index using the controls sample and, for the multivariable analysis, in effect adjusted for the other measure.

**Table 5:** Odds ratio per unadjusted standard deviation^1^ from Dembrower et al. [22].

	Univariable	Multivariable	
DL	1.56 (1.48–1.64)	1.52 (1.42–1.59)	1.55 (1.47–1.64)
Dense area	1.31 (1.24–1.38)	1.15 (1.09–1.22)	-
Percent density	1.18 (1.11–1.25)	-	1.02 (0.95–1.08)

^1^ standard deviation is calculated for each measure adjusted for age and body mass index using the controls sample and, for the multivariable analysis, in effect adjusted for the other measure.

## References

[1] Byng J.W., Yaffe M.J., Jong R.A., Shumak R.S., Lockwood G.A., Tritchler D.L., Boyd N.F. (1998). Analysis of mammographic density and breast cancer risk from digitized mammograms. Radiographics.

[2] Krishnan K., Baglietto L., Stone J., Simpson J.A., Severi G., Evans C.F., MacInnis R.J., Giles G.G., Apicella C., Hopper J.L. (2017). Longitudinal Study of Mammographic Density Measures That Predict Breast Cancer Risk. Cancer Epidemiol. Biomarkers Prev..

[3] Shia W.C., Wu H.K., Huang Y.L., Lin L.S., Chen D.R. (2018). Mammographic Density Distribution of Healthy Taiwanese Women and its Naturally Decreasing Trend with Age. Sci. Rep..

[4] Hopper J.L., Dite G.S., MacInnis R.J., Liao Y., Zeinomar N., Knight J.A., Southey M.C., Milne R.L., Chung W.K., Giles G.G. (2018). Age-specific breast cancer risk by body mass index and familial risk: Prospective family study cohort (ProF-SC). Breast Cancer Res..

[5] Nguyen T.L., Schmidt D.F., Makalic E., Dite G.S., Stone J., Apicella C., Bui M., Macinnis R.J., Odefrey F., Cawson J.N. (2013). Explaining variance in the cumulus mammographic measures that predict breast cancer risk: A twins and sisters study. Cancer Epidemiol. Biomarkers Prev..

[6] Eng A., Gallant Z., Shepherd J., McCormack V., Li J., Dowsett M., Vinnicombe S., Allen S., dos-Santos-Silva I. (2014). Digital mammographic density and breast cancer risk: A case-control study of six alternative density assessment methods. Breast Cancer Res..

[7] Hopper J.L. (2015). Odds per adjusted standard deviation: Comparing strengths of associations for risk factors measured on different scales and across diseases and populations. Am. J. Epidemiol..

[8] Schmidt D.F., Makalic E., Goudey B., Dite G.S., Stone J., Nguyen T.L., Dowty J.G., Baglietto L., Southey M.C., Maskarinec G. (2018). Cirrus: An automated mammography-based measure of breast cancer risk based on textural features. JNCI Cancer Spectrum.

[9] Mavaddat N., Michailidou K., Dennis J., Lush M., Fachal L., Lee A., Tyrer J.P., Chen T.H., Wang Q., Bolla M.K. (2019). Polygenic Risk Scores for Prediction of Breast Cancer and Breast Cancer Subtypes. Am. J. Hum. Genet.

[10] Nguyen T.L., Aung Y.K., Evans C.F., Yoon-Ho C., Jenkins M.A., Sung J., Hopper J.L., Song Y.M. (2015). Mammographic density defined by higher than conventional brightness threshold better predicts breast cancer risk for full-field digital mammograms. Breast Cancer Res..

[11] Nguyen T.L., Aung Y.K., Evans C.F., Dite G.S., Stone J., MacInnis R.J., Dowty J.G., Bickerstaffe A., Aujard K., Rommens J.M. (2017). Mammographic density defined by higher than conventional brightness thresholds better predicts breast cancer risk. Int. J. Epidemiol..

[12] Nguyen T.L., Choi Y.H., Aung Y.K., Evans C.F., Trinh N.H., Li S., Dite G.S., Kim M.S., Brennan P.C., Jenkins M.A. (2018). Breast cancer risk associations with digital mammographic density by pixel brightness threshold and mammographic system. Radiology.

[13] Nguyen T.L., Aung Y.K., Li S., Trinh N.H., Evans C.F., Baglietto L., Krishnan K., Dite G.S., Stone J., English D.R. (2018). Predicting interval and screen-detected breast cancers from mammographic density defined by different brightness thresholds. Breast Cancer Res..

[14] Nguyen T.L., Li S., Dite G.S., Aung Y.K., Evans C.F., Trinh H.N., Baglietto L., Stone J., Song Y.M., Sung J. (2019). Interval breast cancer risk associations with breast density, family history and breast tissue aging. Int. J. Cancer.

[15] Wolfe J.N. (1976). Risk for breast cancer development determined by mammographic parenchymal pattern. Cancer.

[16] Rafferty E.A. (2014). Mammographic breast density: From Wolfe and beyond. Menopause.

[17] Byng J.W., Boyd N.F., Fishell E., Jong R.A., Yaffe M.J. (1994). The quantitative analysis of mammographic densities. Phys. Med. Biol..

[18] Boyd N.F., Guo H., Martin L.J., Sun L., Stone J., Fishell E., Jong R.A., Hislop G., Chiarelli A., Minkin S. (2007). Mammographic density and the risk and detection of breast cancer. N. Engl. J. Med..

[19] Baglietto L., Krishnan K., Stone J., Apicella C., Southey M.C., English D.R., Hopper J.L., Giles G.G. (2014). Associations of mammographic dense and nondense areas and body mass index with risk of breast cancer. Am. J. Epidemiol..

[20] Krishnan K., Baglietto L., Apicella C., Stone J., Southey M.C., English D.R., Giles G.G., Hopper J.L. (2016). Mammographic density and risk of breast cancer by mode of detection and tumor size: A case-control study. Breast Cancer Res..

[21] Wang C., Brentnall A.R., Cuzick J., Harkness E.F., Evans D.G., Astley S. (2018). Exploring the prediction performance for breast cancer risk based on volumetric mammographic density at different thresholds. Breast Cancer Res..

[22] Dembrower K., Liu Y., Azizpour H., Eklund M., Smith K., Lindholm P., Strand F. (2020). Comparison of a Deep Learning Risk Score and Standard Mammographic Density Score for Breast Cancer Risk Prediction. Radiology.

[23] Keller B.M., Chen J., Daye D., Conant E.F., Kontos D. (2015). Preliminary evaluation of the publicly available Laboratory for Breast Radiodensity Assessment (LIBRA) software tool: Comparison of fully automated area and volumetric density measures in a case-control study with digital mammography. Breast Cancer Res..

[24] Wanders J.O.P., van Gils C.H., Karssemeijer N., Holland K., Kallenberg M., Peeters P.H.M., Nielsen M., Lillholm M. (2018). The combined effect of mammographic texture and density on breast cancer risk: A cohort study. Breast Cancer Res..

[25] Dite G.S., MacInnis R.J., Bickerstaffe A., Dowty J.G., Allman R., Apicella C., Milne R.L., Tsimiklis H., Phillips K.A., Giles G.G. (2016). Breast cancer risk prediction using clinical models and 77 independent risk-associated SNPs for women aged under 50 years: Australian breast cancer family registry. Cancer Epidemiol. Biomarkers Prev..

[26] Boyd N.F., Dite G.S., Stone J., Gunasekara A., English D.R., McCredie M.R., Giles G.G., Trichler D., Chiarelli A., Yaffe M.J. (2002). Heritability of mammographic density, a risk factor for breast cancer. N. Engl. J. Med..

[27] Martin L.J., Melnichouk O., Guo H., Chiarelli A.M., Hislop T.G., Yaffe M.J., Minkin S., Hopper J.L., Boyd N.F. (2010). Family history, mammographic density, and risk of breast cancer. Cancer Epidemuiol. Biomakers Prev..

[28] Stone J., Thompson D.J., Dos Santo Silva I., Scott C., Tamimi R.M., Lindstrom S., Kraft P., Hazra A., Li J., Eriksson L. (2015). Novel assocaitions between common breast cancer susceptibility variants and risk-predicting mammographic density measures. Cancer Res..

[29] Lindstrom S., Thompson D.J., Paterson A.D., Li J., Gierach G.L., Scott C., Stone J., Douglas J.A., dos Santos-Silva I., Fernando-Navarro O. (2014). Genome-wide association study identifies multiple loci associated with both mammographic density and breast cancer risk. Nat. Commun..

[30] Pertuz S., Sassi A., Holli-Helenius K., Kamarainen J., Rinta-Kiikka I., Laaperi A.L., Arponen O. (2019). Clinical evaluation of a fully-automated parenchymal analysis software for breast cancer risk assessment: A pilot study in a Finnish sample. Eur. J. Radiol..

[31] Tan M., Mariapun S., Yip C.H., Ng K.H., Teo S.H. (2019). A novel method of determining breast cancer risk using parenchymal textural analysis of mammography images on an Asian cohort. Phys. Med. Biol..

[32] Yala A., Lehman C., Schuster T., Portnoi T., Barzilay R. (2019). A Deep Learning Mammography-based Model for Improved Breast Cancer Risk Prediction. Radiology.

[33] Dench E., Bond-Smith D., Darcey E., Lee G., Aung Y.K., Chan A., Cuzick J., Ding Z.Y., Evans C.F., Harvey J. (2019). Measurement challenge: Protocol for international case-control comparison of mammographic measures that predict breast cancer risk. BMJ Open.

[34] Li S., Wong E.M., Bui M., Nguyen T.L., Joo J.E., Stone J., Dite G.S., Dugue P.A., Milne R.L., Giles G.G. (2019). Inference about causation between body mass index and DNA methylation in blood from a twin family study. Int. J. Obes..

[35] Li S., Dugue P.A., Baglietto L., Severi G., Wong E.M., Nguyen T.L., Stone J., English D.R., Southey M.C., Giles G.G. (2019). Genome-wide association study of peripheral blood DNA methylation and conventional mammographic density measures. Int. J. Cancer.

[36] Glasziou P.P., Jones M.A., Pathirana T., Barratt A.L., Bell K.J. (2019). Estimating the magnitude of cancer overdiagnosis in Australia. Med. J. Aust..

[37] Hopper J.L. (2017). Genetics for population and public health. Int. J. Epidemiol..

